# First experience with 0.31 Tesla low-field MRI in post-mortem fetal examinations

**DOI:** 10.1007/s00414-025-03698-6

**Published:** 2025-12-26

**Authors:** Dominic Gascho, Anna Kuntze, Eva Deininger-Czermak, Christian Ottow, Volker Vieth, Peter Barth, Andreas Schmeling, Tobias Krähling

**Affiliations:** 1 Institute of Forensic Medicine, University of Zurich, Zurich, Switzerland; 2 Institute of Pathology, University and University Hospital Münster, Münster, Germany; 3 Clinic of Radiology, University and University Hospital Münster, Münster, Germany; 4 Clinic of Radiology and Neuroradiology, Ibbenbüren Hospital, Ibbenbüren, Germany; 5 Institute of Legal Medicine, University and University Hospital Münster, Münster, Germany

**Keywords:** Pediatric pathology, Perinatal death, Postmortem MRI, Postmortem imaging, Virtual autopsy, Virtopsy

## Abstract

Post-mortem imaging has become an important adjunct or alternative to autopsy in perinatal pathology and forensic medicine, particularly when conventional autopsy is limited or declined. However, high-field MRI, currently the post-mortem imaging gold standard for fetuses, is not always available in many forensic settings. The aim of this study was to evaluate whether a 0.31 Tesla low-field MRI scanner, which offers advantages in affordability, installation, infrastructural requirements such as reduced power, cooling, and shielding demands, and lower safety constraints associated with its field strength, can deliver diagnostically relevant information in post-mortem fetal examinations within a forensic context. Three fetuses underwent post-mortem low-field MRI prior to autopsy and histopathological assessment. Imaging findings were compared with autopsy results to evaluate diagnostic concordance and potential added value in a forensic context. Low-field MRI successfully identified key anatomical abnormalities: Fetus #1 showed a cerebral lesion on MRI that could not be recognized during the autopsy due to autolysis-related loss of structural integrity of the brain after opening the skullcap. Fetus #2 presented with a left-sided diaphragmatic hernia with enterothorax. Fetus #3 exhibited gastroschisis and a renal abnormality. Using the 0.31 Tesla low-field MRI scanner provided sufficient diagnostic detail to support and complement the forensic examinations of fetal deaths. It can improve the documentation and interpretation of findings in perinatal forensic cases, enhance diagnostic certainty in decomposed or fragile remains, and offer a viable alternative when parental refusal or legal constraints prevent full autopsy.

**Supplementary Information:**

The online version contains supplementary material available at 10.1007/s00414-025-03698-6.

## Introduction

Post-mortem imaging is increasingly recognized as a valuable noninvasive approach for investigating deaths in children, offering an alternative or complement to traditional autopsy methods [1–5]. However, significant variability exists among facilities regarding the availability of imaging modalities and the extent of services provided [6].

Radiography remains the most widely used and resource-efficient post-mortem imaging technique. However, its inherent diagnostic limitations, such as poor soft tissue contrast, often necessitate the use of more advanced imaging modalities, including computed tomography (CT) and magnetic resonance imaging (MRI) [7]. Post-mortem CT is particularly applicable for forensic investigations due to its ability to rapidly visualize skeletal structures, gas or air accumulations, major hemorrhages, and foreign bodies. Nevertheless, its limited soft tissue contrast and the lack of any possibility for physical distribution of contrast enhancement post-mortem renders it insufficient for accurately determining the cause of death in fetuses, neonates, and infants, where subtle pathological changes are often present [7, 8].

In contrast, post-mortem MRI has emerged as a more promising tool for investigating pediatric deaths, especially in children under one year of age [9]. Its superior soft tissue contrast enables the identification of major pathological conditions, including brain abnormalities, congenital anomalies, and organ-specific diseases [7]. Despite its diagnostic advantages, post-mortem MRI is not widely adopted due to several challenges, including limited accessibility to MRI facilities and the high costs associated with acquiring and maintaining standard high-field MRI scanners. Given the small size of fetuses, neonates, and infants, an opportunity exists to explore the feasibility of utilizing more resource-efficient low-field MRI scanners for post-mortem examinations. Low-field MRI is commonly used for extremity imaging in clinical practice or for forensic age estimation [10]. It may provide a practical alternative for paediatric post-mortem imaging by offering sufficient soft tissue contrast while reducing operational costs and logistical barriers, with the understanding that its use as an alternative is undertaken in full awareness of its inherent technical limitations.

This study aims to assess whether a small low-field MRI scanner, originally designed for extremity imaging, can serve as a viable alternative for post-mortem examinations of fetuses. The article discusses the scanner’s diagnostic capabilities, image quality, and practical implementation, with the goal of determining its potential role in enhancing post-mortem fetal investigations while improving accessibility and resource-efficiency.

## Materials and methods

### Pathologic examination

Ethical approval was obtained by the responsible ethics committee (University of Muenster, 2023-270-f-S). In all three cases, medically indicated pregnancy termination was performed in the University Hospital Muenster due to sonographic abnormalities detected during routine prenatal examinations. Maternal consent was secured for the further examination of three fetuses.

Fetal examinations were performed at the Gerhard-Domagk-Institute of Pathology, Muenster, by two pathologists following standard protocols. External inspection including photography, weight, and length as well as radiological evaluation, were conducted prior to MRI. For internal examination, organs were exenterated, fixed in 4% paraformaldehyde, and assessed for weight and size. Tissue samples from major organs (heart, lungs, liver, kidneys, spleen, thymus, thyroid, lymph nodes, intestine, reproductive organs, umbilical cord) were processed for histology. The brain was fixed in 4% paraformaldehyde after opening the skullcap and examined at the Institute of Neuropathology, Muenster.

### MR scanner and sequences

All three fetuses were examined on a 0.31 Tesla O-scan system for extremities (Esaote S.p.A., Genoa, Italy) using a dedicated dual phased array (DPA) coil for the knee joint prior to autopsy and histological examination. The MR imaging protocol included a T1-weighted spin-echo sequence (SE T1), a dual-echo fast spin-echo sequence with proton density- (PD) and T2-weighting (Fast PD-T2), a proton density-weighted spin-echo sequence in Dixon technique (SPED PD) and a work-in-progress T2 mapping sequence based on two 3D gradient echo sequences (3D SHARC, Self-Harmonized Advanced Reconstruction Concept) with different flip angles. For Fetus #1, additional T1- and T2-weighted 3D steady-state gradient echo sequences (3D SST1 and 3D SST2) were acquired. The sequence parameters are shown in detail in Tables 1 and 2.

**Table 1:** MRI protocol 2D sequences

	SE T1	Fast PD-T2	SPED PD
Plane	axial	axial	axial
Echo time TE (ms)	20	30/120	20
Repetition time TR (ms)	470	6500	1800
Slice thickness (mm)	3	3	3
Slice gap (mm)	0.3	0.3	0.3
Field of view (mm x mm)	160 × 160	160 × 160	160 × 160
Acquired matrix size	256 × 256	256 × 256	256 × 256
Acquired pixel size (mm x mm)	0.63 × 0.63	0.63 × 0.63	0.63 × 0.63
Recon pixel size (mm x mm)	0.63 × 0.63	0.63 × 0.63	0.63 × 0.63
Number of averages	2	1	1
SpeedUp	120	120	140
Scan time (mm: ss)	13:36	12:34	11:10

**Table 2:** MRI protocol 3D sequences

	3D T2map	3D SST1	3D SST2
Plane	axial	axial	axial
Echo time TE (ms)	10	12	10
Repetition time TR (ms)	20	24	20
Flip angle (°)	30/60	30	50
Field of view (mm x mm x mm)	200 × 200 × 160	160 × 160 × 160	160 × 160 × 160
Acquired matrix size	192 × 192 × 116	192 × 192 × 116	192 × 192 × 116
Acquired voxel size (mm x mm x mm)	1.04 × 1.04 × 1.38	0.83 × 0.83 × 1.38	0.83 × 0.83 × 1.38
Recon voxel size (mm x mm x mm)	0.78 × 0.78 × 0.78	0.63 × 0.63 × 0.63	0.63 × 0.63 × 0.63
Number of averages	1	1	1
SpeedUp	120	120	120
Scan time (mm: ss)	11:12	07:25	06:51

MRI examination of the fetuses were performed between 2 and 6 days after the medical pregnancy termination (fetus #1: 3 days, fetus #2: 6 days, and fetus #3: 2 days). Until MR examination, the fetuses were stored at 4 °C, whereas the MRI examination itself was conducted at room temperature without additional temperature control. The fetuses were positioned inside the knee coil on a self-constructed table mounted on a phantom holder provided by the manufacturer. While the field-of-view (FOV) of the knee coil (max. imaging FOV of 140 mm) was sufficient to image the entire fetus #1, the examination for fetuses #2 and #3 was divided into two acquisitions with intentionally overlapping coverage. The first acquisition included the head and the entire thorax, while the second acquisition started superior to the heart and therefore covered the lower thorax together with the abdomen and lower body down to the knees. This resulted in a partial overlap in the thoracic region between the two segments. All images were reviewed by a board-certified radiologist with more than five years of experience in post-mortem forensic imaging.

## Results

### Study population

Fetus #1 was from 13th week of gestation with a weight of 12 g (reference value according to Ludwig [11] for 13 weeks of gestation: 60 g, *note: no standard deviation is provided for this developmental stage in the cited literature*), a crown-heel length of 8.1 cm and a crown-rump length of 5.9 cm. Fetus #2 was from 26th week of gestation with a weight of 540 g (reference value according to Ludwig [11] for 26 weeks of gestation: 663 g, SD: 227 g), a crown-rump length of 19.2 cm and a crown-heel length of 27.8 cm. Fetus #3 was from 21st week of gestation with a weight of 223 g (reference value according to Ludwig [11] for 21 weeks of gestation: 353 g, SD: 125 g), a crown-heel length of 23.6 cm, and a crown-rump length of 17.1 cm. The complete reports of the external examinations are provided in the supplementary materials.

### MRI examination

**Fig. 1:**
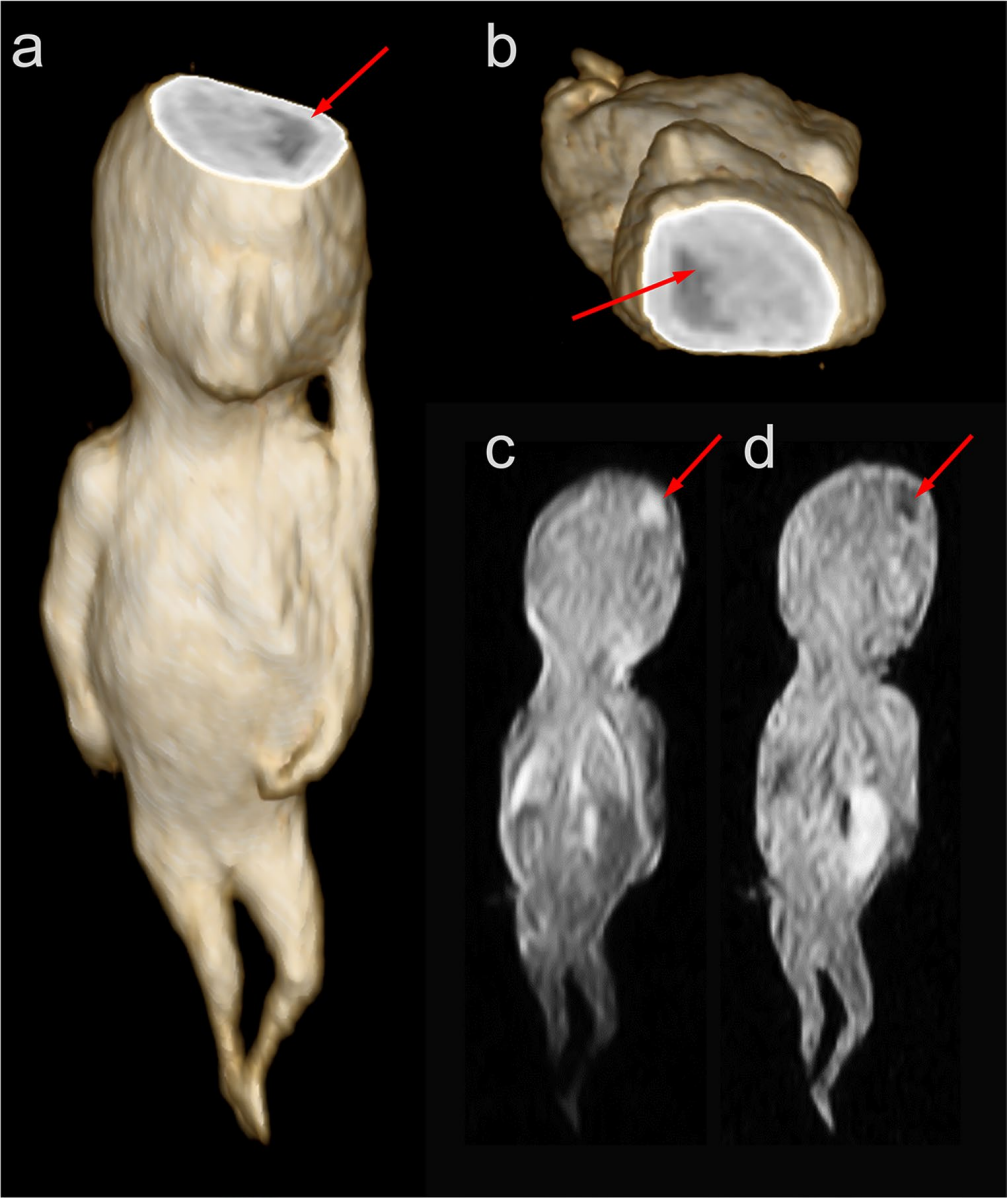
Volume rendering of fetus #1 with a virtual cut through the brain illustrates a cerebral finding in the left parietal brain (**a** and **b**). The cerebral finding is hyperintense (bright) on T2-weighted MRI (**c**) and a hypointense (dark) on T1-weighted MRI (**d**)

**Fig. 2:**
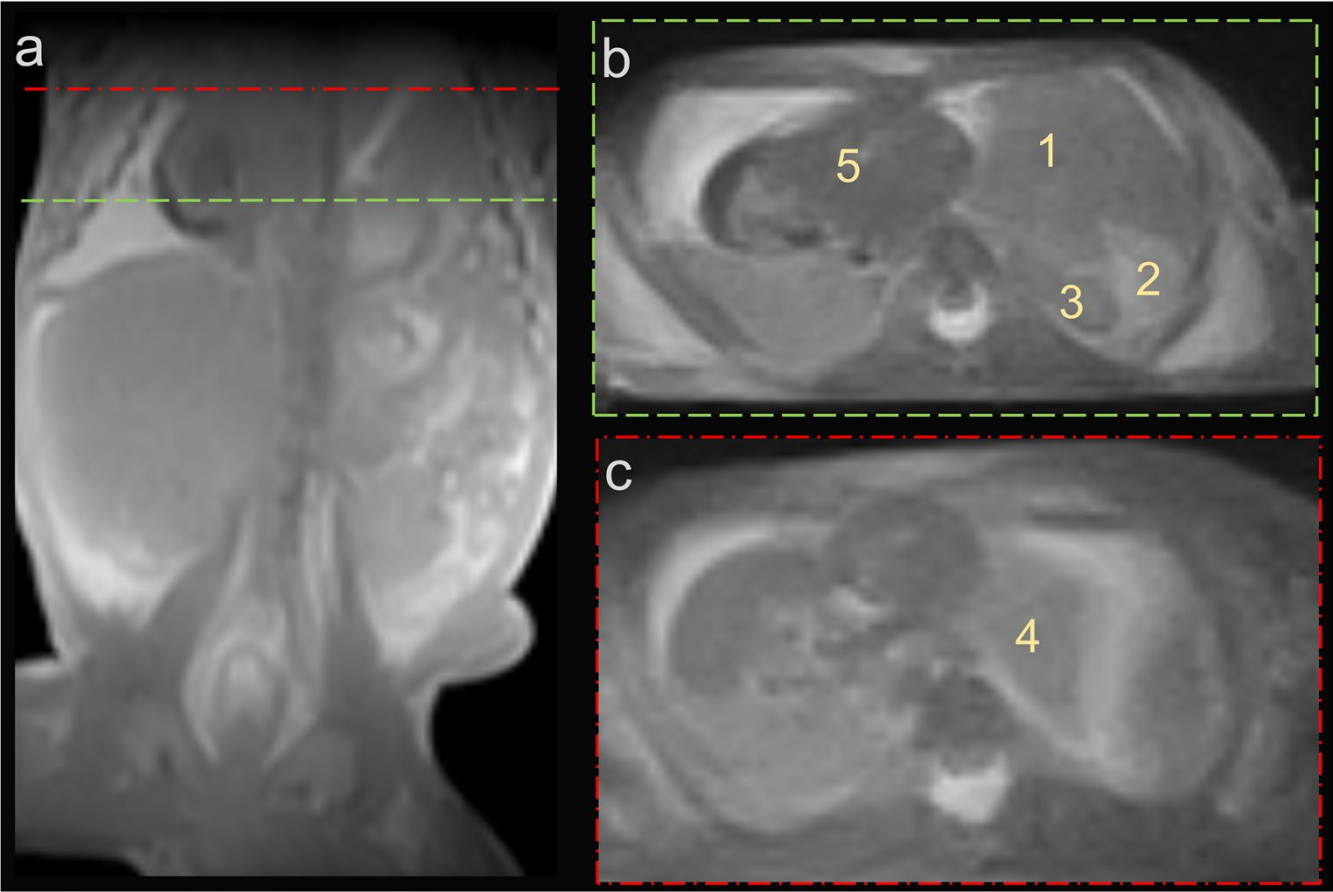
Fetus #2 presented with a left-sided diaphragmatic hernia (a) characterized by displacement of the liver (1), spleen (2), and intestinal loops (3) into the left hemithorax, along with left lung hypoplasia (4) and a rightward shift of the heart (5)

**Fig. 3:**
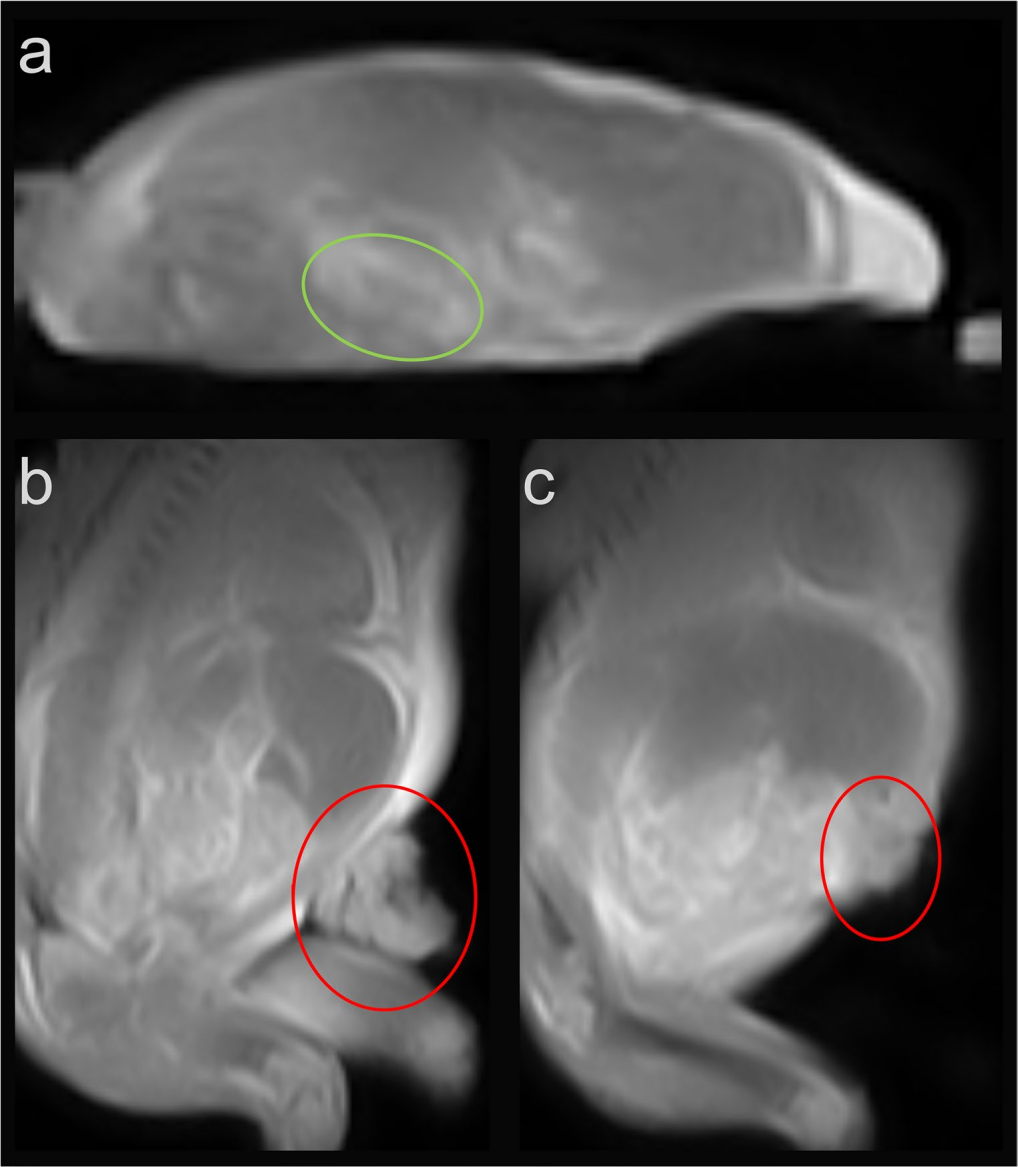
Fetus #3 demonstrated a suspected oligocystic kidney (**a**, green circle), as well as a defect due to gastroschisis with herniated bowel (**b** and **c**, red circle)

On MRI, fetus #1 exhibited a hyperintense (bright) signal in the left parietal brain region on T2-weighted imaging and a hypointense (dark) signal on T1-weighted imaging (Fig. 1). Fetus #2 presented with a left-sided diaphragmatic hernia, characterized by displacement of the liver, spleen, and intestinal loops into the left hemithorax, along with left lung hypoplasia and a rightward shift of the heart *(*Fig. 2). Fetus #3 demonstrated a suspected oligocystic kidney, as well as a defect due to gastroschisis with herniated bowel (Fig. 3). In all three examinations, the interpreting radiologist subjectively perceived 3D sequences as more helpful than 2D sequences for identifying findings and assessing small fetal structures. This reflects qualitative experience rather than a systematic comparison. Based on these impressions, 3D sequences were found to be advantageous because isotropic imaging with multiplanar reconstruction improved the evaluation of complex malformations.

### Internal autopsy and additional examinations

The autopsy of fetus #1 revealed hydrops fetalis with generalized edema and hemorrhagic ascites. A liver capsule hematoma and small parenchymal lacerations were considered procedure-related. Histology showed organ development appropriate for gestational age. Severe autolysis resulted in partial liquefaction and loss of structural integrity of the brain during skullcap opening, limiting neuropathological assessment; within these limits, cytoarchitecture appeared normal without hypoxic or inflammatory changes. Chromosomal analysis confirmed Edwards’ syndrome (trisomy 18). The autopsy of fetus #2 confirmed a left-sided diaphragmatic hernia with enterothorax, left lung hypoplasia, polysplenia, and a single umbilical artery. Incomplete colonic rotation was present, with the appendix located near the liver. Histology showed delayed left lung development (pseudoglandular stage); other organs were age-appropriate. Brain autolysis again limited neuropathology but multifocal recent intracerebral hemorrhages were identified. Chromosomal analysis demonstrated a terminal deletion of chromosome 15q. A clinically suspected ventricular septal defect was not confirmed. In fetus #3, a duplicated spleen and a markedly enlarged left kidney with cystic areas were found, histologically consistent with oligocystic renal dysplasia. The brain was not examined per maternal request.

## Discussion

The post-mortem examinations of three fetuses utilizing a 0.31 Tesla MRI scanner, originally designed for extremity imaging, demonstrate that such a system can noninvasively detect relevant pathological findings. These results indicate that low-field MRI could serve as a valuable complementary imaging tool in fetal autopsies, enabling visualization of structural abnormalities, such as brain malformations, pleural or abdominal effusions, and hernias, that are likely to be disrupted during exenteration. Having MRI results available prior to autopsy could aid in localizing specific findings, allowing for targeted section and subsequent histopathological examination. As the low-field MRI scanner used in this study does not have an automatically movable table, the limitation of the FOV dimensions requires examinations of larger fetuses to be performed in several sections, which must be positioned manually. This means that it is not possible to combine the individual bed positions into corresponding whole-body series without extensive post-processing.

In fetus #1, MRI demonstrated a cerebral abnormality that could not be evaluated at autopsy or during subsequent neuropathological examination because the brain was severely autolyzed with partial liquefaction and loss of structural integrity, limiting morphological assessment. This discrepancy highlights a well-known limitation of fetal autopsy, namely that macroscopic assessment of the brain is particularly challenging due to its early post-mortem autolysis. Post-mortem MRI, in contrast, allows for in situ visualization of cerebral structures and revealed a cerebral finding. However, a major challenge in post-mortem fetal MRI is the interpretation of cerebral findings. Post-mortem changes, including autolysis, tissue degradation, and fluid shifts, can mimic or obscure pathological processes. Furthermore, MRI contrast is temperature-dependent, meaning that image quality and contrast can vary significantly based on the post-mortem interval and cooling conditions. MRI sequences, particularly T1-weighted, are temperature sensitive because tissue relaxation times and the diffusivity of water molecules depend on temperature. Despite these challenges, it is noteworthy that even in a fetus weighing only 12 g and at an early stage of development, the low-field MRI scanner achieved an acceptable spatial resolution. Although optimized for extremity imaging, the system provided sufficient image quality to demonstrate a cerebral finding in the post-mortem examination of a very small fetus weighing only 12 g. In fetus #2, the primary pathological finding, a left-sided diaphragmatic hernia, was clearly visualized on post-mortem MRI. This finding underscores the adequacy of image quality in terms of both spatial and contrast resolution for detecting structural pathology such as herniation. Given that congenital diaphragmatic hernia, depending on its severity, is a life-threatening condition, the ability of post-mortem MRI to accurately depict this defect supports its potential role in forensic and perinatal pathology. For fetus #3, MRI successfully indicated an enlarged left kidney and clearly delineated the abdominal wall defect due to gastroschisis, with the herniated bowel loops distinctly visible.

The findings of this study align with broader discussions in the literature regarding the role of post-mortem MRI in fetal and perinatal imaging. Shelmerdine and Arthurs [12] reviewed post-mortem perinatal imaging, emphasizing MRI’s strength in detecting structural abnormalities and supporting its role as a valuable adjunct to fetal autopsy, particularly when macroscopic examination is challenging. While post-mortem MRI is highly effective in identifying major structural abnormalities, histological confirmation remains essential for definitive diagnosis [13]. Furthermore, post-mortem MRI has limitations in diagnosing infections and metabolic disorders [12]. These limitations could potentially be addressed by magnetic resonance spectroscopy, although its post-mortem application remains limited to date [14]. The low-field MRI scanner used in this study does not currently have this examination option.

A Europe-wide survey by Whitby et al. [15] highlighted the variability in post-mortem MRI practices and the logistical challenges restricting its routine use. In this context, accessibility refers to the practical feasibility of implementing MRI services for post-mortem imaging rather than to the current distribution of scanners. Low-field MRI systems may alleviate several barriers associated with high-field scanners, including higher costs, increased power and cooling requirements, more extensive shielding and stricter safety constraints, and may therefore facilitate the establishment of post-mortem imaging services in resource-limited environments. However, the survey also underscored the necessity of standardized protocols to ensure diagnostic consistency, a crucial consideration for integrating low-field MRI into routine practice.

High-resolution microfocus CT has also been explored as an imaging option for very small fetal specimens [16], but it requires contrast-agent staining, involves prolonged preparation times and alters tissue integrity to a degree that precludes subsequent autopsy or histological assessment, which limits its usefulness in routine forensic workflows.”

## Conclusion

In conclusion, the present study demonstrates that low-field MRI can be a useful and resource-efficient approach for post-mortem fetal imaging. Importantly, the detection of a cerebral abnormality in fetus 1, which could not be assessed or confirmed at autopsy due to pronounced autolysis causing fragmentation of the brain, illustrates a key advantage of post-mortem MRI. In situ visualization of the brain allows structural anomalies to be depicted that may otherwise be obscured or destroyed during autopsy. The relatively low acquisition and running costs of permanent-magnet low-field systems further strengthen their potential as a complementary or alternative method in perinatal pathology. Future research should focus on comparing low-field MRI with high-field MRI and histopathology to determine its diagnostic accuracy, optimizing the MRI sequences for post-mortem imaging and particularly for soft tissue differentiation, and evaluating its application in forensic settings, where noninvasive imaging could serve as an alternative when parental consent for autopsy is limited. Assessing larger case series is needed to further establish feasibility and clinical utility. Overall, low-field MRI scanners presents an innovative and resource-efficient approach for post-mortem fetal imaging, offering new opportunities in perinatal pathology, forensic medicine, and pediatric radiology.

## Supplementary Information

Below is the link to the electronic supplementary material.

Supplementary Material 1

## Declarations

This study was approved by the Ethics Committee of the University of Muenster, Germany (Reference: 2023-270-f-S). All procedures performed in this study involving human participants were carried out in accordance with the ethical standards of the institutional and/or national research committee and with the 1964 Declaration of Helsinki and its later amendments or comparable ethical standards.
