# Supplementary material for: First experience with 0.31 Tesla low-field MRI in post-mortem fetal examinations
Source: Int J Legal Med. 2025 Dec 26;140(3):1435–41. doi: 10.1007/s00414-025-03698-6 (PMC13160973; doi:10.1007/s00414-025-03698-6)
Supplement: Supplementary file 1 — Supplementary Material 1 [file 414_2025_3698_MOESM1_ESM.pdf]

Electronic supplementary material

–

External examination and illustrated autopsy findings

First Experience with 0.31 Tesla Low-Field MRI in Post-Mortem Fetal Examinations

International Journal of Legal Medicine (*Electronic ISSN 1437-1596*)

Dominic Gascho[1*], Anna Kuntze[2*], Eva Deininger-Czermak[1], Christian Ottow[3], Volker Vieth[3,4], Peter Barth[2], Andreas Schmeling[5], Tobias Krähling[3]

[1] Institute of Forensic Medicine, University of Zurich, Zurich, Switzerland

[2] Institute of Pathology, University and University Hospital Münster, Münster, Germany

[3] Clinic of Radiology, University and University Hospital Münster, Münster, Germany

[4] Clinic of Radiology and Neuroradiology, Ibbenbüren Hospital, Ibbenbüren, Germany

[5] Institute of Legal Medicine, University and University Hospital Münster, Münster, Germany

*shared first author

Corresponding author: dominic.gascho@irm.uzh.ch

Fetus #1

Extrenal examination: The external genitalia appeared undifferentiated. The interpupillary distance measured 0.5 cm. The nose and mouth were unremarkable, with no evidence of cleft formation, and the tongue appeared normal. The skin was largely intact but exhibited edematous changes and small hemorrhages. The umbilical cord included three vessels and showed no signs of infection. An omphalocele was present and comprised small parts of the intestine. Each hand and foot had five distinct digits, the feet displayed a deformity of the calcaneus, called “Rocker bottom feet”. Radiological evaluation, as far as assessable, indicated a normally developed axial skeleton.

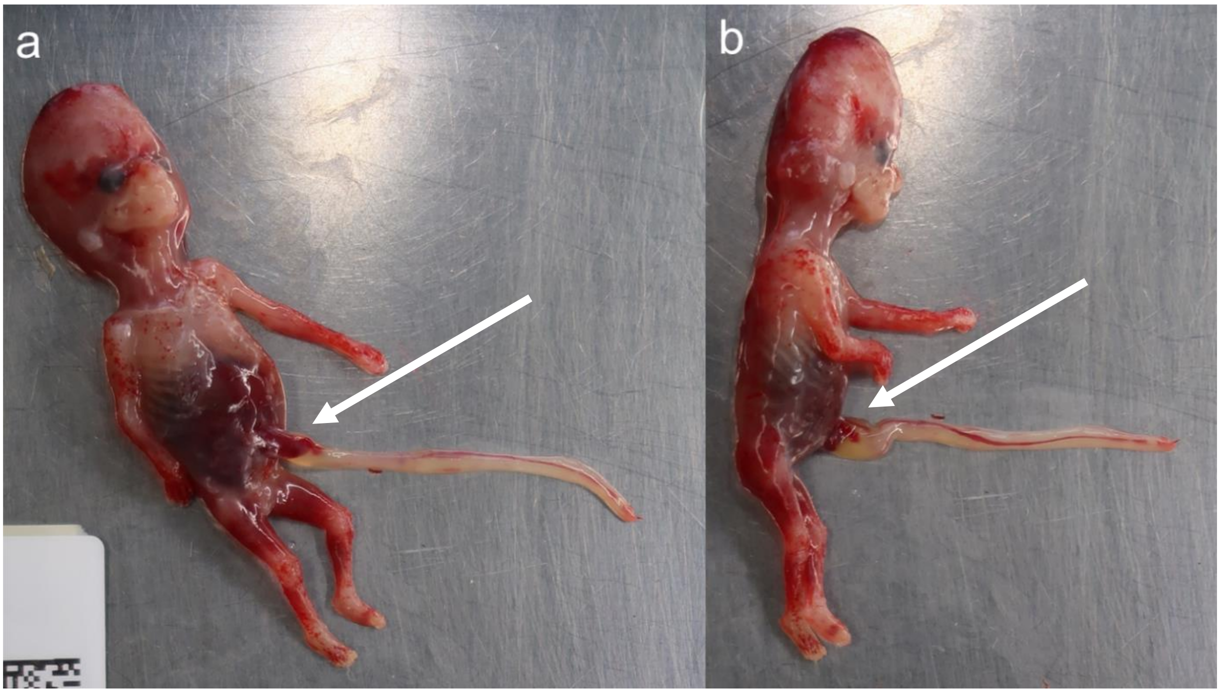

Fetus #1 presented a convex plantar surface with a prominent heel (“Rocker bottom feet”) and a small omphalocele (a and b, arrow).

Fetus #2

External examination: The fetus was phenotypically male. The interpupillary distance measured 1.4 cm. Both eyelids could be separated. The nose was normally formed. The ears were low-set and flattened. No cleft lip, jaw, or palate was detected. The tongue appeared normal. The skin was fragile, with a reddish-purple hue and easy detachment due to beginning autolysis. No lanugo hair was observed. The umbilical cord included two vessels and showed no signs of infection. Testicular descent was incomplete. The anus was freely probe-patent. The hands appeared broad and stubby, without a four-finger crease. The feet were edematous. Radiologically, five rays were present in both hands and feet, with no apparent skeletal malformations.

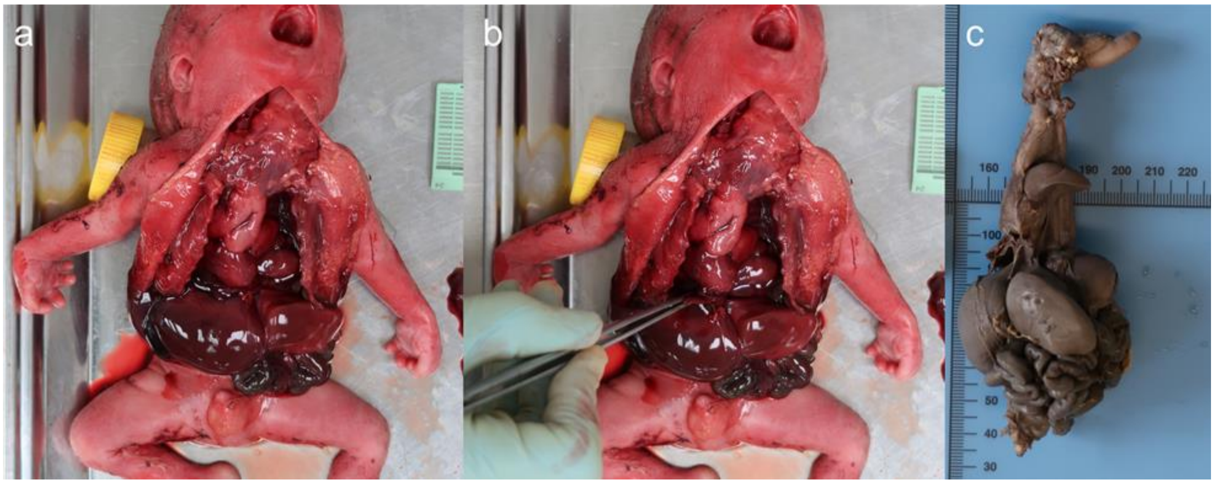

Fetus #2: internal examination of showing left-sided diaphragmatic hernia with enterothorax and consecutive left lung hypoplasia (a). Forceps point to the diaphragmatic defect (b). Organs after formalin-fixation (c).

Fetus #3

External examination: The fetus was phenotypically female. The interpupillary distance measured 1.3 cm. The ears were low-set. No cleft formations were present, and the tongue appeared normal. A postmortem skin defect (2.8 × 2.6 cm) was noted on the ventral neck and thorax, exposing the cervical musculature. The eyelids could be separated but were closed, with no distinguishable irises or pupils due to beginning autolysis. The umbilical cord included three vessels and showed no signs of infection. A gastroschisis was observed, with a right-sided abdominal wall defect located above the umbilical cord, through which the prolapsed large intestine and terminal ileum were visible, including the appendix vermiformis in its typical location. Radiological evaluation, as far as assessable, indicated a normally developed axial skeleton.

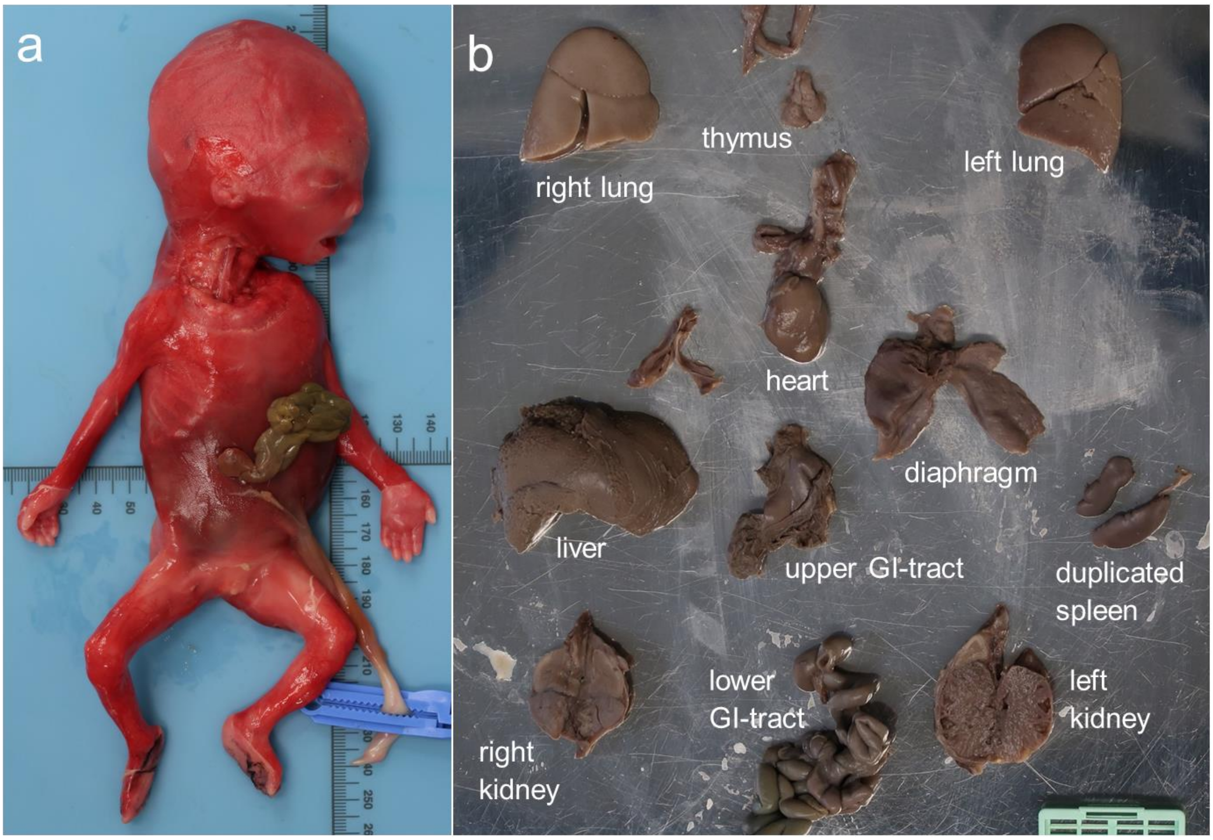

Fetus #3 with gastroschisis (a) Fixed organs of Fetus #3 (b): cut surfaces of both kidneys with adherend adrenal glands, showing diffuse enlargement and cystic alteration of the left kidney; a duplicated spleen was also present. Note: the lower genitourinary tract is not depicted.
